# Myasthenia gravis

**DOI:** 10.1186/1750-1172-2-44

**Published:** 2007-11-06

**Authors:** Vern C Juel, Janice M Massey

**Affiliations:** 1 Division of Neurology, Box 3403, Duke University Medical Center, Durham, North Carolina, 27710, USA

## Abstract

Myasthenia gravis (MG) is a rare, autoimmune neuromuscular junction disorder. Contemporary prevalence rates approach 1/5,000. MG presents with painless, fluctuating, fatigable weakness involving specific muscle groups. Ocular weakness with asymmetric ptosis and binocular diplopia is the most typical initial presentation, while early or isolated oropharyngeal or limb weakness is less common. The course is variable, and most patients with initial ocular weakness develop bulbar or limb weakness within three years of initial symptom onset. MG results from antibody-mediated, T cell-dependent immunologic attack on the endplate region of the postsynaptic membrane. In patients with fatigable muscle weakness, the diagnosis of MG is supported by: 1. pharmacologic testing with edrophonium chloride that elicits unequivocal improvement in strength; 2. electrophysiologic testing with repetitive nerve stimulation (RNS) studies and/or single-fiber electromyography (SFEMG) that demonstrates a primary postsynaptic neuromuscular junctional disorder; and 3. serologic demonstration of acetylcholine receptor (AChR) or muscle-specific tyrosine kinase (MuSK) antibodies. Differential diagnosis includes congenital myasthenic syndromes, Lambert Eaton syndrome, botulism, organophosphate intoxication, mitochondrial disorders involving progressive external ophthalmoplegia, acute inflammatory demyelinating polyradiculoneuropathy (AIDP), motor neuron disease, and brainstem ischemia. Treatment must be individualized, and may include symptomatic treatment with cholinesterase inhibitors and immune modulation with corticosteroids, azathioprine, cyclosporine, and mycophenolate mofetil. Rapid, temporary improvement may be achieved for myasthenic crises and exacerbations with plasma exchange (PEX) or intravenous immunoglobulin (IVIg). Owing to improved diagnostic testing, immunotherapy, and intensive care, the contemporary prognosis is favorable with less than five percent mortality and nearly normal life expectancy.

## Disease name

Myasthenia gravis, Autoimmune myasthenia gravis

### Included diseases

Autoimmune myasthenia gravis (MG) encompasses all of the immunologically-mediated disorders affecting the endplate region of the postsynaptic neuromuscular junction. Nearly all of these disorders involve a loss of immunological self-tolerance, though transitory neonatal MG is a self-limited disorder that follows passive transfer of maternal antibodies to the fetus. Congenital myasthenic syndromes stem from genetic mutations that result in abnormal neuromuscular transmission.

MG is termed ocular MG when weakness is exclusive to the eyelids and extraocular muscles, and generalized MG when weakness extends beyond these ocular muscles. Seropositive (SP) MG defines disease with circulating antibodies to the acetylcholine receptor (AChR), while seronegative (SN) patients lack these antibodies. Recently, antibodies to muscle-specific tyrosine kinase (MuSK) have been demonstrated in over 40% of patients with generalized, SN MG [1-5].

## Definition and diagnostic criteria

MG remains one of the most challenging medical diagnoses due to its fluctuating character and to the similarity of its symptoms to those of other disorders. Although a formal clinical classification system and research standards have been established for MG, [6] there are no widely accepted formal diagnostic criteria. The most important elements of diagnosis are clinical history and examination findings of fluctuating and fatigable weakness, particularly involving extraocular and bulbar muscles. A clinical diagnosis may be confirmed by laboratory testing including: 1. pharmacologic testing with edrophonium chloride that elicits unequivocal improvement in strength; 2. electrophysiologic testing with repetitive nerve stimulation (RNS) studies and/or single-fiber electromyography (SFEMG) that demonstrates a primary postsynaptic neuromuscular junctional disorder; or 3. by serological demonstration of AChR or MuSK antibodies.

## Epidemiology

Although MG is rare, prevalence rates for MG have increased over time, likely due to improvements in diagnosis and treatment. Recent prevalence rates approach 20/100,000 [7]. A wide range of incidence is reported with an estimate of about 2.0 to 10.4/million/year in Virginia [8] to 21.27/million/year in Barcelona, Spain [9]. The onset of MG is influenced by gender and age in a bimodal fashion. In patients younger than 40, women predominate with a ratio of 7:3. In the fifth decade, new cases of MG are evenly distributed between men and women. After age 50, new cases of MG are slightly more common in men with a ratio of 3:2 [10,11].

Pediatric MG is very rare. Juvenile MG is an autoimmune disorder, while congenital MG results from genetic mutations that impair neuromuscular transmission. Transient neonatal MG is a self-limited disorder related to placental antibody transfer in maternal autoimmune MG. It may be difficult to make the distinction between juvenile MG and congenital MG, particularly in the absence of AChR or MuSK antibodies, or a clear history of ptosis and other manifestations of hypotonia from the time of birth that would suggest genetic disease. These issues are discussed in depth by Andrews [12].

## Clinical description

In MG, patients present with fluctuating and fatigable weakness of specific muscle groups rather than with generalized fatigue or pain. The weakness is variable from day to day and from hour to hour, but it is generally worse later in the day. Sustained exercise and increased body temperature may increase the degree of weakness. Ocular weakness with asymmetric ptosis and binocular diplopia is the most common initial presentation, while early or isolated oropharyngeal or limb weakness is less common.

Ocular weakness presents as fluctuating, fatigable, and sometimes alternating ptosis and binocular diplopia that resolves with closing or covering one eye. Many patients report difficulties with driving, reading, or watching television. Bright lights may be quite bothersome. Retrospectively, many patients report periods of intermittent blurred vision before they were able to discern dual visual images. Examination may demonstrate asymmetrical weakness of multiple extraocular muscles that cannot be attributed to a single cranial neuropathy. Pupillary function is normal. Ptosis may be elicited or increased with sustained upgaze. In MG, ptosis is generally asymmetrical, and it may be associated with ipsilateral frontalis muscle contraction to help compensate for the weak levator palpebrae. Excessive lid elevation or Cogan's lid twitch sign may be observed when gaze is directed from down to upward.

Dysthyroid (Graves) ophthalmopathy may coexist with ocular MG. Although external ophthalmoplegia may occur in either disorder, dysthyroid ophthalmopathy produces proptosis, not ptosis, owing to enlarged extraocular muscles. The enlarged muscles may be demonstrated by orbital magnetic resonance imaging (MRI).

Jaw closure muscles are frequently affected in MG, but strength is usually normal in jaw opening muscles. Patients may complain of difficulty in chewing candy or tough meats, and some modify their diet to compensate for this difficulty. Some patients assume a thoughtful resting posture by placing the thumb beneath the chin in order to hold the jaw closed. The jaw closure muscles can be examined by exerting several seconds of sustained downward pressure on the chin while the patient attempts to hold the jaw closed.

Many patients exhibit a depressed or expressionless facial appearance. Actions such as whistling, using straws, or inflating balloons may be impaired. A "myasthenic snarl" may be observed when the patient attempts to smile. The snarl follows contraction of the middle portion of the upper lip while the upper mouth corners fail to contract. On examination, many patients exhibit weak forced eye closure that can easily be overcome by the examiner. Bell's phenomenon with upward and lateral rotation of the eyes on attempted closure is observed when the examiner defeats a patient's forced eye closure. Patients with mild lower facial weakness develop a transverse pucker when they attempt to hold air within inflated cheeks. With more overt lower facial weakness, air readily escapes through the lips when the cheeks are squeezed. In severe lower facial weakness, the lips cannot be voluntarily opposed.

Oropharyngeal muscle weakness produces dysarthria and dysphagia. With weakness of palatal muscles, nasal speech develops as air escapes through the nose. This may become increasingly apparent with prolonged speaking. Liquid may also escape through the nose during attempted swallowing with nasal regurgitation. Myasthenic weakness of laryngeal muscles is associated with a hoarse, breathy voice. Incomplete glottic closure during swallowing may produce aspiration. Examination may reveal reduced or absent palate elevation. Tongue weakness may be demonstrated when the patient attempts to protrude either cheek with the tongue against the resistance of the examiner's finger applied to the cheek. With marked tongue weakness, the patient may be unable to protrude the cheek in the absence of applied resistance by the examiner. With severe lingual weakness, the tongue may not protrude beyond the lips. When myasthenic tongue weakness is chronic, tongue atrophy and triple furrowing may develop with accentuated median and lateral lingual furrows.

Neck flexor and extensor muscles are often weak in MG. Though the neck flexors are usually weaker, a "dropped head syndrome" due to neck extensor muscle weakness may occur. Although painless weakness is the rule in MG, patients with neck extensor weakness may experience posterior neck myalgias.

Limb weakness in MG may be associated with complaints of difficulty performing overhead tasks with the arms, and there may be difficulty climbing stairs due to lower extremity weakness. Examination reveals asymmetrical weakness involving any muscle group in the limbs, though the deltoids, triceps brachii, wrist and finger extensors, and foot dorsiflexors are often involved.

## Etiology

Autoimmune MG results from antibody-mediated, T cell-dependent immunologic attack on the postsynaptic membrane of the neuromuscular junction. Abnormal neuromuscular transmission and clinical weakness in MG result from the effects of antibodies that bind to various epitopes of the skeletal muscle endplate region. In most cases, antibodies bind to the main immunogenic region of the α-subunit of the AChR, though MG patients with antibodies to MuSK exhibit clinical weakness and electrophysiologic findings that are quite similar to MG patients with AChR antibodies. MuSK initiates aggregation of AChRs at the muscle endplate during development, [2] but the function of MuSK in mature skeletal muscle and the pathophysiology of MG related to MuSK antibodies are currently unknown.

In SP MG, binding of antibody to the AChR initiates autoimmune attack on the endplate region. Subsequent damage to the postsynaptic membrane results in simplification of the normal, highly-infolded surface that is accompanied by reduced number and density of AChR [13]. The functional loss of AChRs reduces the probability of successful neuromuscular transmission following quantal release of acetylcholine by the motor nerve terminal, resulting in clinical weakness in striated muscles.

MG and other autoimmune disorders result from the loss of tolerance to self-antigens. T-lymphocyte tolerance to self-antigens is established in the thymus, and thymic abnormalities are often present in MG. Thymic hyperplasia is observed in about 65% of MG patients, and thymomas are present in about 10% of MG patients [14]. MG patients with thymoma have more severe and generalized weakness, higher AChR antibody titers, and more severe electrophysiologic abnormalities. Accordingly, patients with SN and ocular MG are less likely to have thymomas. Most thymic tumors are benign, well differentiated, and encapsulated. Patients with MG should undergo chest computed tomography (CT) to exclude the presence of thymoma. While thymoma resection is necessary to prevent compromise of mediastinal structures, the benefit of thymectomy for patients with non-thymomatous MG remains uncertain.

## Diagnostic methods

### Pharmacologic testing

#### Edrophonium testing

Edrophonium chloride is an acetylcholinesterase inhibitor with rapid onset (approximately 30 seconds) and short duration (approximately 5 minutes) of pharmacologic action. Edrophonium chloride temporarily improves the safety factor of neuromuscular transmission and may elicit improved strength in patients with abnormal neuromuscular transmission. Edrophonium testing is considered positive when unequivocal improvement in strength follows intravenous administration of edrophonium. Development of increased weakness may also suggest abnormal neuromuscular transmission. The primary limitation of edrophonium testing relates to selection of an objective muscle strength parameter for assessment. Therefore, edrophonium testing is most useful in patients who have significant ptosis or restricted extraocular movements that can be graded objectively. In other muscles, volition and the muscarinic effects of edrophonium may complicate strength measurement and render the test uninterpretable.

The sensitivity of edrophonium testing has been estimated to be about 86% for ocular MG and 95% for generalized MG [15]. False positive edrophonium testing may occur in other neurological conditions including lower motor neuron disorders and brainstem tumors [16-19].

During testing, up to 10 mg of intravenous edrophonium chloride may be administered. Because of the potential for serious muscarinic side effects including bronchospasm and bradycardia, atropine should be readily available. Typical muscarinic side effects include increased sweating, lacrimation, salivation, nausea, and diarrhea. An incremental dosing schedule should be utilized with one minute observation periods following each dose of edrophonium. If muscle strength improves clearly within one minute following any dose increment, the test is considered to be positive and the procedure is concluded. This strategy reduces the risk of giving excessive edrophonium and producing untoward muscarinic side effects. An initial 2 mg dose and subsequent doses of 2 mg, 3 mg, and 3 mg are given if needed.

### Electrophysiologic testing

#### RNS studies

With low rates of motor nerve stimulation (2–5 Hz), RNS depletes the immediate stores of acetylcholine at the neuromuscular junction. This reduces the safety factor and probability of successful neuromuscular transmission. In neuromuscular junction disorders, the safety factor is reduced, and further reduction by RNS causes some endplate potentials to fail to reach depolarization threshold. This results in a failure to elicit muscle fiber action potentials. With a reduced number of individual muscle fiber action potentials, the compound muscle action potential (CMAP) becomes reduced in both amplitude and area with a resulting decremental response.

In MG, RNS study findings are abnormal when the amplitude of the fourth CMAP is reduced more than 10% from the baseline value. This may not be present in stimulus trains recorded following rest, but it may only develop in trains collected subsequent to an exercise period as a consequence of postactivation exhaustion. The sensitivity of RNS is increased when recordings are made from clinically weak muscles. Careful attention to proper technique is important to avoid erroneous results. There must be adequate immobilization of the stimulating and recording electrodes, delivery of supramaximal stimuli, muscle warming to 35°C, and withholding of acetylcholinesterase inhibitors for at least 12 hours prior to testing. In general, proximal muscles including facial muscles, trapezius, deltoid, and biceps brachii are more likely to exhibit abnormal findings. In MG, when RNS studies are performed in a hand and in a shoulder muscle, the overall sensitivity is approximately 60%. RNS studies are relatively more sensitive in generalized MG and relatively less sensitive in ocular MG [20].

#### SFEMG

SFEMG is the most sensitive diagnostic test for detecting abnormal neuromuscular transmission. In SFEMG, individual muscle fiber action potentials generated by the same motor neuron are recorded by a specialized concentric needle with a 25 **μ**m diameter recording surface and a 500 Hz high-frequency filter. In most normal muscles, this arrangement facilitates recordings from two individual muscle fiber action potentials. The variability in time interval between the firing of one muscle fiber potential with relation to the other is termed the neuromuscular jitter [21].

SFEMG should be performed in a clinically weak muscle whenever possible. In many laboratories, the extensor digitorum communis (EDC) is studied initially. If the findings are normal in the EDC, a facial muscle should be studied [22]. When a facial and a limb muscle are studied, SFEMG is over 97% sensitive for detecting MG [23]. A finding of normal jitter in a clinically weak muscle virtually excludes MG as a cause of weakness in that muscle. However, SFEMG also demonstrates abnormal neuromuscular transmission related to other motor unit disorders including motor neuropathic and myopathic disorders. Normal SFEMG fiber density measurements can aid in distinguishing primary disorders of neuromuscular transmission from other motor unit disorders such as motor neuropathic or myopathic processes. In light of its reduced specificity, SFEMG must be performed and interpreted in the appropriate clinical context to avoid false positive results due to diseases other than those primarily affecting the neuromuscular junction. It is a time-consuming test that requires special expertise and equipment that are not available in all centers.

### Serological testing

#### AChR antibodies

The AChR binding antibody assay utilizes purified human skeletal AChRs incubated with patient serum immunoglobulin. The assay is very specific, and positive antibody studies confirm MG in a patient with appropriate symptoms and clinical findings. AChR binding antibodies are present in approximately 80% of patients with generalized MG, but in only 55% of patients with ocular MG [24,25]. About one-half of prepubertal children with MG are SN [26]. Though relatively less sensitive than SFEMG, AChR binding antibodies are highly specific for autoimmune MG. Rarely, false positive results in AChR binding antibody assays have been observed in patients with other autoimmune diseases such as systemic lupus erythematosus, rheumatoid arthritis, and inflammatory neuropathy. False positive results have also been reported in motor neuron disease, patients with thymoma without MG, and relatives of patients with MG [27]. Some initially SN patients may seroconvert within the first several months of disease. Seroconversion may be identified in these patients by repeating the AChR binding antibody studies after six months of symptoms [28].

The AChR modulating antibody assay measures the degradation rate of labeled AChRs from cultured human myotubes. AChR blocking antibodies compete for the acetylcholine binding site or allosterically inhibit binding of radiolabelled **α**-bungarotoxin to the AChR [27]. The AChR modulating and blocking antibody assays are probably useful only when the AChR binding antibody assay is negative, since they increase diagnostic sensitivity only slightly. Approximately 4% of patients with negative AChR binding antibodies have an elevated AChR modulating antibody assay, and approximately 1% of patients with negative AChR binding antibodies demonstrate increased AChR blocking antibodies [29].

#### Anti-striated muscle antibodies

Anti-striated muscle or anti-striational antibodies react with contractile elements of skeletal muscle. They are found in 30% of patients with adult-onset MG, and they appear to be more common in patients with later disease onset [30]. These antibodies may be useful as a serological marker for thymoma in younger patients. Anti-striated muscle antibodies have been demonstrated in 80% of patients with thymoma in the absence of MG. Following thymoma resection, a rise in anti-striated muscle antibody titer may suggest recurrent tumor [30]. In one series, thymomas were demonstrated in 60% of patients with anti-striated muscle antibodies and MG beginning before age 50, and in less than 2% of patients without these antibodies [31].

#### MuSK antibodies

Antibodies to MuSK have been demonstrated recently in about one third of patients with generalized SN MG. Patients with MuSK MG are predominantly female and may exhibit prominent bulbar, neck, shoulder girdle, and respiratory weakness [1,2,4]. MuSK appears to facilitate clustering of AChR at the end plate region in the developing neuromuscular junction, though the role of MuSK antibodies in producing disease at mature neuromuscular junctions has not yet been defined.

#### Other antibodies

Antibodies against the intracellular skeletal muscle protein titin may be present in patients with thymoma, but they are also present in about half of patients with late-onset MG without thymoma [32,33]. Ryanodine antibodies are also associated with late-onset MG. Patients with ryanodine antibodies may exhibit severe, treatment-resistant MG associated with malignant thymomas [34]. Although the role for these antibodies in the diagnosis of MG has yet to be determined, they may have prognostic value and expedite chest imaging studies for detection of thymoma.

### Other testing

Chest computerized tomography (CT) should be performed in patients with MG to exclude the presence of thymoma. Chest CT is more sensitive than plain chest radiographs for delineating anterior mediastinal masses, and chest MRI does not improve diagnostic sensitivity. Iodinated contrast agents have rarely precipitated significant worsening of myasthenic weakness [35,36]. Though this is an uncommon phenomenon [37], we do not routinely use iodinated contrast agents during chest CT studies performed to assess for thymoma. Since MG often co-exists with other autoimmune disorders, particularly autoimmune thyroid disease, patients should undergo thyroid function testing along with testing for other autoimmune disorders when clinically appropriate.

### Summary

Pharmacologic testing with intravenous edrophonium is sensitive when performed in patients with significant ptosis or external ophthalmoparesis. RNS studies may demonstrate impaired neuromuscular transmission, especially when performed recording from clinically weak muscles, though they are relatively insensitive in ocular and in mild generalized MG. SFEMG is the most sensitive laboratory test for MG, although abnormal SFEMG findings may be seen in motor neuropathic and in myopathic disorders. Normal SFEMG findings in a clinically weak muscle exclude a diagnosis of MG. In the clinical context of fluctuating and fatigable weakness, AChR or MuSK antibodies confirm the diagnosis of MG, though nearly half of patients with ocular MG are SN.

## Differential diagnosis

Differential diagnosis includes other disorders of the neuromuscular junction including Lambert Eaton syndrome, botulism, congenital myasthenic syndromes, and tick paralysis. In addition, acute inflammatory demyelinating polyradiculoneuropathy (AIDP) and variants of AIDP affecting cranial muscles such as the Miller-Fisher and cervical-brachial-pharyngeal variants may simulate MG, though the weakness does not have the same variability. Mitochondrial neuromuscular disorders, particularly those featuring external ophthalmoplegia and ptosis, may simulate MG. However, the onset of symptoms is gradual, and the weakness does not fluctuate greatly. Motor neuron disease involving oropharyngeal weakness may appear similar to MG, though the presence of corticobulbar features and increased fiber density measurements on SFEMG can assist in distinguishing these entities. Finally, brainstem ischemia may simulate the fluctuating character of MG, though unlike MG, consciousness, balance and coordination, and sensation are often impaired.

## Management

Treatment must be individualized to each patient with MG. The overall goal is to reestablish or to approximate normal clinical neuromuscular function while minimizing adverse effects. Few treatments have been subjected to rigorous, prospective, placebo-controlled study in MG. Factors to be considered in selecting treatment include the distribution, duration, and severity of myasthenic weakness and functional impairment, the risk for treatment complications related to medical comorbidities, age, and gender, and the ability of the patient to obtain medication and comply with drug dosing schedules and toxicity monitoring. In general, the increased risks related to long-term immune modulation become more acceptable in more severe MG to offset increased morbidity and mortality related to uncontrolled disease. A detailed review of treatment issues in autoimmune MG may be found elsewhere [38].

### Acetylcholinesterase inhibitors

Acetylcholinesterase inhibitors slow the hydrolysis of acetylcholine at the neuromuscular junction and provide temporary improvement in strength in many patients with MG. Although acetylcholinesterase inhibitors were among the earliest and remain one of the most widely prescribed treatments for MG, there are no controlled clinical trials of these agents in MG. Acetylcholinesterase inhibitors are a symptomatic therapy for MG and do not retard the underlying autoimmune attack on the neuromuscular junction. The roles for acetylcholinesterase inhibitors in MG include treatment of ocular and mild generalized disease, treatment in patients who cannot receive immune suppression, and adjunctive treatment for patients receiving immunotherapy with residual or refractory myasthenic weakness.

Effective dosing of acetylcholinesterase inhibitors reduces myasthenic weakness, minimizes muscarinic medication side effects, and must be individualized to each patient's distribution of weakness and diurnal symptom fluctuation. For example, patients with prominent dysphagia may benefit by taking pyridostigmine 30 minutes before meals and those with more symptoms in the afternoon and evening may shift their dosing to later in the day to better target symptoms. Pyridostigmine bromide is generally better tolerated than neostigmine bromide due to fewer gastrointestinal side effects. A long-acting form of pyridostigmine bromide is available, though it is absorbed irregularly and tends to be overdosed. Initial dosing of pyridostigmine bromide is usually 30 mg three times a day and may be advanced to 90 mg three to four times a day. Improvement in strength begins about 20 to 30 minutes after ingestion. Peak improvement is usually observed at about 45 minutes after ingestion, and benefits may last four hours or more.

The most salient side effects of acetylcholinesterase inhibitors relate to increased muscarinic activity and include nausea, vomiting, abdominal cramping, diarrhea, diaphoresis, and increased lacrimation, salivation, and bronchial secretions. These dose dependent and self-limited side effects may be treated with glycopyrrolate, and the gastrointestinal side effects may be treated with diphenoxylate hydrochloride with atropine or loperamide hydrochloride.

Cholinergic crisis may develop with excessive dosing of acetylcholinesterase inhibitors in patients with more severe MG. In cholinergic crises, depolarization blockade at diseased neuromuscular junctions results in increased weakness, and increased muscarinic activity generates copious oropharyngeal and bronchial secretions that may obstruct the airway or be aspirated. Signs of cholinergic crisis include weakness indistinguishable from myasthenic weakness, muscle fasciculations, and symptoms of increased muscarinic activity including bradycardia.

### Corticosteroids

Corticosteroids are the most widely used immune modulating agents for MG. Although the mechanism of action in MG is unknown, corticosteroids have numerous effects on the immune system including reduction of cytokine production [39]. Corticosteroids are often used as the initial immunotherapy in patients with ocular and generalized MG, particularly in patients with unsatisfactory responses to acetylcholinesterase inhibitors. These agents may produce rapid improvement in MG, though they are often associated with the liability of significant dose-dependent side effects and occasionally elicit transient and potentially serious myasthenic exacerbations within the first two weeks of treatment. Prednisone treatment produced significant improvement in strength within two to three weeks in retrospective studies of MG [40,41], and a Cochrane review cites significant short term benefit in MG with corticosteroids [42]. Marked improvement or remission was achieved in 80% MG patients in one large series with a 3.1 month mean time to marked improvement and a median time to maximum benefit between five and six months [40].

The most reliable clinical responses to corticosteroids occur with a high-dose daily regimen that is gradually tapered based on clinical improvement in strength. The initial prednisone dose is typically 60–80 mg/day or 1.5–2.0 mg/kg/day. This initial dose is maintained for two to four weeks, and strength is reassessed. If strength is definitely improved, dosing is transitioned to an alternate day schedule of 100–120 mg/alternate day to minimize adrenal suppression. Occasional patients are unable to tolerate an alternating-day regimen due to mood instability, variation in MG, or difficult glycemic control in occasional diabetic patients.

Myasthenic relapses may be delayed for three weeks after reductions in corticosteroid dosing, and rapid tapering may precipitate myasthenic exacerbations or crises. Therefore, corticosteroid tapering must be slow, judicious, and preceded by clinical reassessment of strength. Patients are reassessed at four to eight week intervals, and if they have maintained or improved strength and no recurrent symptoms, the prednisone dose is tapered by 10 mg/alternate day to 30 mg/alternate day, and then by 5 mg/alternate day to 20 mg/alternate day. Subsequent tapering by 2.5 mg/alternate day should be performed over longer intervals of at least 12 weeks, since many patients will begin to experience recurrent symptoms in this dosing range unless they are being treated with another immune modulating agent.

The adverse effects of corticosteroids are numerous, well known, and largely dose-dependent. These include: hypertension, fluid retention, weight gain, potassium loss, hyperlipidemia, diabetes mellitus, osteoporosis, gastric ulceration, cataracts, glaucoma, moon facies, obesity, acne, skin friability, juvenile growth suppression, and mood/personality changes. Individuals at particular risk for side effects include those who are diabetic or glucose intolerant, obese, hypertensive, osteoporotic or post-menopausal, and those with affective or thought disorders. An alternative immune modulator may be considered in such patients.

A high protein, low fat, low carbohydrate, low sodium diet is recommended to prevent untoward weight gain, hyperlipidemia, and fluid retention. Serum electrolytes, glucose, blood pressure, and weight are monitored periodically during treatment. To minimize osteopenia, calcium carbonate 1500 mg/day and vitamin D 600 IU/day are recommended. In post-menopausal women, a baseline bone density evaluation is performed and repeated every six months during treatment. Before initiating treatment with corticosteroids or any long-term immunotherapy, PPD testing should be considered as a screen for tuberculosis.

Corticosteroid-related MG exacerbations may produce transient but potentially serious increases in myasthenic weakness in up to 15% of patients beginning treatment with corticosteroids [40]. The increased weakness develops within 7–10 days after treatment begins and may last for up to one week before strength improves [40,43]. Patients at greatest risk for morbidity related to this phenomenon are those with more severe bulbar or generalized weakness and/or compromised respiratory function. When beginning corticosteroid treatment in such patients, strength and respiratory function should be closely monitored. Plasma exchange (PEX) may be performed prior to starting corticosteroids to circumvent or minimize corticosteroid-related MG exacerbations. In such cases, corticosteroids are initiated after a clear improvement in strength attributable to PEX is documented.

An alternative dosing strategy of starting corticosteroids at a low initial dose with gradual dose increases has been advocated to reduce the risk for corticosteroid-related MG exacerbations [44]. However, this strategy does not eliminate the risk for exacerbation [45], and onset of strength improvement is less predictable and may be significantly delayed.

### Azathioprine

Azathioprine is hepatically converted to 6-mercaptopurine, an active anti-metabolite that blocks nucleotide synthesis and T-lymphocyte proliferation. Azathioprine is an effective agent for long-term immune modulation in MG as a steroid sparing drug or as initial immunotherapy. Compared to corticosteroids, azathioprine has a favorable side effect profile for long-term use. However, the typically long delay of four to eight months from beginning treatment with azathioprine to improved strength in MG is a significant liability to its usefulness, particularly in MG patients with progressive disease or functionally limiting symptoms.

In a prospective, randomized, double-blind study comparing prednisolone with prednisolone plus azathioprine, the prednisolone plus azathioprine treatment group exhibited longer remissions, fewer treatment failures, fewer side effects, and reduced maintenance doses of prednisolone [46]. The initial dose is 50 mg/day is increased by 50 mg/day every week to a target therapeutic dose of 2–3 mg/kg/day.

Side effects include dose dependent myelosuppression with macrocytic anemia, leukopenia, and thrombocytopenia, toxic hepatitis, and alopecia. Hypersensitivity pancreatitis represents a rare, but serious idiosyncratic reaction, and patients with sustained abdominal pain taking azathioprine should be screened with serum amylase and lipase assays. With long-term use, there is a small increased risk for lymphoma [47].

Azathioprine is potentially teratogenic, and women of child bearing potential using azathioprine should practice effective contraception. An idiosyncratic allergic reaction with rash, fever, nausea, vomiting, and abdominal pain occurs in 10–15% patients within the first three weeks of treatment [48,49]. The reaction resolves within one day of stopping azathioprine, and will recur if the patient is rechallenged with the drug.

Monitoring for myelosuppression is recommended with weekly blood count and liver transaminase measurements weekly for the first month of treatment, then monthly for the first year, then every three months thereafter unless the dosage is increased. Erythrocyte macrocytosis is expected and acceptable within the therapeutic dosage range. If white blood cell count (WBC) falls below 3500/mm^3^, the dosage should be reduced, and if WBC falls below 3000/mm^3^, azathioprine should be discontinued.

### Cyclosporine

Cyclosporine exerts an immunomodulatory effect by blocking interleukin-2 production and T lymphocyte proliferation. Although effective, the use of cyclosporine in MG has been limited by its nephrotoxicity and numerous drug interactions. In light of this, cyclosporine is used in MG as a steroid-sparing agent or for refractory generalized disease. After therapeutic levels of cyclosporine are achieved and maintained, improvement in strength is usually observed within two months.

In a population of steroid-dependent patients with MG, a randomized, double blind, placebo-controlled study of cyclosporine demonstrated significantly improved strength in the cyclosporine treatment group [50]. In a long-term retrospective study, 96% MG patients improved with cyclosporine treatment, and steroids could be tapered or discontinued in 95% patients [51]. The typical cyclosporine dose is 2.5 mg/kg every 12 hours to achieve a target serum trough level of 100–150 microgram/L.

Side effects of cyclosporine include hypertension, nephropathy, tremor, hirsutism, gingival hypertrophy, headaches, and nausea. Accordingly, relative contraindications to cyclosporine include poorly controlled hypertension, renal insufficiency or failure, malignancy, and inability to comply with blood monitoring or medication precautions.

Periodic monitoring is necessary to achieve therapeutic trough cyclosporine levels and to prevent nephrotoxicity. Assessments of blood pressure, serum creatinine and the trough serum cyclosporine level should be performed frequently until a stable, therapeutic dose of cyclosporine is achieved and after new medications are begun that have the potential to interact with cyclosporine.

Cyclosporine has problematic interactions with numerous drugs that may result in nephrotoxicity, significant increases in serum drug levels, and/or significant increases or decreases in serum cyclosporine levels [52]. The most common cyclosporine interactions and potential complications include non-steroidal anti-inflammatory agents with impaired renal function, angiotensin converting enzyme inhibitors eliciting hyperkalemia, and HMG CoA reductase inhibitors precipitating cholesterol-lowering agent myopathy.

### Mycophenolate mofetil

Mycophenolate mofetil (MMF) is a relatively novel immune modulator that selectively inhibits T and B lymphocyte proliferation by blocking purine synthesis exclusively in lymphocytes. In human kidney transplant trials, MMF exhibited minimal toxicity [53]. Given a paucity of side effects, MMF is used in MG both as a steroid-sparing agent and as initial immunotherapy in patients at risk for corticosteroid complications. Improved strength is observed within about two months after reaching a therapeutic dose of MMF.

Significantly improved strength in MG patients taking MMF has been demonstrated in a retrospective case series [54], in an open label pilot study in steroid dependent or refractory MG [55], and in a double-blind placebo control pilot trial in MG [56].

However, in a recently concluded multi-center, randomized, controlled trial of low dose prednisone versus low dose prednisone with MMF, there was no clinically significant benefit in MG patients treated with MMF combined with low dose prednisone beyond that observed in MG patients treated with low dose prednisone only during the initial three month trial period. Analysis of the open label phase of this study is currently pending [57].

The standard MMF dosage in MG is 1000–1500 mg twice a day. Higher doses are associated with myelosuppression, and periodic blood counts are performed during treatment as surveillance against leukopenia or anemia. There is a small increased risk for lymphoproliferative disorders in transplant patients, and a case of central nervous system (CNS) lymphoma has been documented in a patient with MG after three years of MMF treatment [58]. Side effects are relatively mild. In one series, diarrhea, abdominal pain, and nausea were reported in 27% patients, though only 6% patients discontinued MMF due to these side effects [59].

### Plasma exchange

PEX is used in MG to achieve rapid, temporary improvement in strength. During PEX, plasma containing AChR antibodies is separated from whole blood and replaced by albumin or fresh frozen plasma. The procedure requires catheterizing large caliber veins. Although many patients have large antecubital veins that may be accessed serially for PEX procedures, some patients require placement of large bore dual lumen central venous catheters. PEX is generally reserved for short-term treatment to achieve rapid strength improvement in myasthenic exacerbations or crises, to prepare patients for thymectomy or another surgical procedure, to prevent steroid-related MG exacerbations in susceptible patients beginning corticosteroid treatment, and for rare patients refractory to all other treatments. An National Institutes of Health (NIH) consensus statement supports use of PEX in these instances [60]. Although there have been no controlled clinical trials of PEX in MG, several series demonstrate significantly improved strength in most patients with severe MG undergoing PEX [61-63]. PEX trials in MG are summarized in a Cochrane review [64].

Typically, a PEX series consists of five to six exchanges of two to three liters on alternate days. Most PEX complications are related to issues of vascular access, particularly to complications of large bore central venous catheters. Excessive fluid volume shifts during the procedure may result in hypotension or fluid overload and congestive heart failure. Sepsis and hypotension are contraindications to PEX.

Improved strength is generally observed after the second or third exchange procedure in most MG patients. Unless another form of treatment is employed, the improved strength is temporary and lasts only a few weeks at best.

### Intravenous immunoglobulin

IVIg has been utilized in a number of autoimmune neuromuscular disorders including acute and chronic inflammatory polyneuropathy. It is thought to act by down regulation of autoantibodies and/or induction of anti-idiotypic antibodies. In MG, IVIg may provide short-term improvement in strength for MG exacerbations and crises, for surgical preparation in patients who are poor PEX candidates because of vascular access issues, and in patients with septicemia.

A number of studies demonstrate efficacy for IVIg in MG. A small randomized, controlled trial of IVIg 1.2 and 2.0 gm/kg compared with PEX in MG patients with exacerbation or crisis showed comparable efficacy between the PEX and IVIg treatment groups [65]. Another retrospective multicenter study demonstrated that PEX was better than IVIg in ability to successfully extubate patients in crisis at two weeks and in functional outcome at one month [66]. However, the PEX groups in both studies sustained more cardiovascular and infectious complications. Although the magnitude of responses appears to be comparable between PEX and IVIg, treatment failures have been reported to IVIg with subsequent response to PEX [67]. IVIg trials in MG are summarized in a Cochrane review [68]. The time to improved strength following IVIg infusions is somewhat variable, as demonstrated by a trial of IVIg given to prepare MG patients for thymectomy in which maximal response was delayed by up to 19 days [69]. IVIg is generally administered as 10% solution, and the standard dosage is 2 gm/kg over two to five days. However, one randomized trial found no added benefit for doses of 2 gm/kg over 1 gm/kg for MG exacerbations [70]. Pretreatment with acetaminophen and diphenhydramine may reduce the frequency and severity of idiosyncratic reactions.

Side effects include volume overload, particularly for patients with cardiomyopathy or valvular heart disease, solute-induced renal failure, especially in patients with preexisting renal insufficiency or diabetic nephropathy [71], and idiosyncratic reactions such as fever, chills, nausea, vomiting, vascular headaches, and aseptic meningitis. High infusion rates may be associated with thrombosis and stroke [72]. Serum immunoglobulin quantitation to screen for IgA deficiency is recommended, since IVIg preparations contain traces of IgA.

### Thymectomy

Thymectomy has been widely performed in an effort to achieve medication-free remission in MG following Blalock's early observations of remissions following thymectomy in non-thymomatous MG [73,74]. To date, there have been no prospective, randomized studies completed to assess the technique or effectiveness of thymectomy in non-thymomatous MG. Fortunately, a large, international multicenter trial is currently enrolling subjects to assess the benefit of thymectomy in non-thymomatous MG [75,76]. An evidence-based review was recently performed to address the role of thymectomy in the management of MG, and outcomes of thymectomy in controlled, nonrandomized studies were systematically reviewed [77]. Although patients undergoing thymectomy in non-thymomatous MG were more likely to achieve medication-free remission, become asymptomatic, or exhibit clinical improvement, the association between thymectomy and improved outcomes could attribute either to thymectomy or to differences in the study populations. Therefore, in non-thymomatous MG, thymectomy should be considered as an option to increase the probability of remission or improvement [77]. The response to thymectomy is not immediate and may be delayed for several years [78-80].

Controversies related to thymectomy in non-thymomatous MG include the ideal timing of the procedure with respect to MG onset, course, and patient age, the optimal surgical technique, whether patients with exclusively ocular MG should undergo thymectomy, and whether patients with SN or MuSK MG benefit from thymectomy.

Because of increased surgical risk and reduced life span, thymectomy is rarely performed in patients at greater than age 60 years for non-thymomatous MG. There is evidence to suggest that the best clinical responses occur if thymectomy is performed early in the course of MG [81], though this may attribute to a non-linear remission rate [77], as remission is more likely to occur shortly after diagnosis rather than with more chronic disease [82]. The role for thymectomy in non-thymomatous ocular MG is also uncertain [83,84]. In MuSK MG, no thymomas have been reported to date, and findings in recent clinical series raise doubt about benefits of thymectomy in MuSK MG patients [1,2,4].

Thymectomy may be performed via several approaches. Though more invasive than other approaches, a combined transsternal-transcervical technique is considered to be optimal as it provides the widest exposure for complete removal of thymic tissue that may be widely distributed in mediastinum and neck [85]. Surgical techniques for thymectomy have been reviewed by Jaretzki and colleagues [86]. Preoperative PEX or IVIg is performed to improve strength in patients with moderate or severe generalized or bulbar MG or in patients with MG-related respiratory insufficiency [87]. In contemporary series of thymectomy for MG, mortality is less than 1%. Complications include respiratory failure due to myasthenic crisis in 6%, infection in 11%, and recurrent laryngeal or phrenic nerve injury in 2% [88].

## Prognosis

Most patients develop initial symptoms of extraocular muscle weakness with asymmetric ptosis and diplopia. The course is frequently variable, particularly within the first year of disease. Nearly 85% of patients with initial ocular symptoms progress to develop weakness of bulbar and limb muscles within the first three years [10,89]. Initial presentations with oropharyngeal and limb weakness are less common. Maximum disease severity is reached within the first year in almost two-thirds of patients [11]. Myasthenic crisis, or respiratory failure due to myasthenic weakness occurs in about 20% of patients, usually within the first year of illness [90,91]. Myasthenic symptoms and signs may worsen in the setting of systemic illness, particularly viral upper respiratory infections, thyroid disease, pregnancy, increased body temperature, and exposure to drugs that impair neuromuscular transmission (Table 1) [23,92].

**Table 1:** Medications that may exacerbate weakness in myasthenia gravis

D-penicillamine
Curare and related drugs
Selected antibiotics including aminoglycosides (tobramycin, gentamycin, kanamycin, neomycin, streptomycin), macrolides (erythromycin, azithromycin, telithromycin [Ketek^®^]), and fluoroquinolones (ciprofloxacin, norfloxacin, ofloxacin, pefloxacin)
Quinine, quinidine or procainamide
Beta-blockers
Calcium channel blockers
Magnesium salts (milk of magnesia, some antacids, tocolytics)
Botulinum toxin

Early in the course of MG, symptoms may fluctuate and occasionally remit, although such remissions are rarely permanent [89,93]. Three major stages of generalized MG have been proposed [94]. An active stage characterized by relapses and remissions lasting approximately seven years is followed by an inactive stage lasting about 10 years. The inactive stage is characterized by less disease volatility, though patients may experience exacerbations related to intercurrent illnesses, pregnancy, or exposure to medications that compromise neuromuscular transmission. In the ultimate stage of "burned-out" disease, untreated weakness may become fixed in association with muscle atrophy.

Prior to the widespread use of immunomodulators, prognosis for patients with MG was grim with about 30% mortality [89]. Along with advances in mechanical ventilation and intensive care, immunotherapy has been one of the major factors contributing to improved outcome in MG, and contemporary disease-specific mortality is less than 5% [95].

## Unresolved questions

There are several contemporary issues related to MG that remain to be resolved. Issues pertaining to MuSK MG include determining the pathophysiologic role of MuSK antibodies in the development of MG, whether the immunological attack on the endplate region in MuSK MG is similar to that of SP MG, and whether thymectomy benefits patients with MuSK MG. The benefit of thymectomy in non-thymomatous SP MG is poorly defined at present, and an international, multicenter trial is currently being undertaken to determine whether and to what degree thymectomy is beneficial in non-thymomatous disease [76]. Whether corticosteroid treatment begun early in the course of ocular MG can prevent generalization has yet to be demonstrated. Finally, it remains to be determined whether and under what circumstances can immune modulation be discontinued without significant risk of relapse in MG patients that have achieved remission with immunotherapy.
